# *Notes from the Field:* Understanding Smoke Exposure in Communities and Fire Camps Affected by Wildfires— California and Oregon, 2020

**DOI:** 10.15585/mmwr.mm6949a4

**Published:** 2020-12-11

**Authors:** Kathleen Navarro, Ambarish Vaidyanathan

**Affiliations:** ^1^Division of Field Studies and Engineering, National Institute for Occupational Safety and Health, CDC; ^2^Division of Environmental Health Science and Practice, National Center for Environmental Health, CDC.

During 2020, the United States has experienced unseasonably higher fire activity than in past years, resulting in >7.8 million burned acres; as of December, wildfires were still active in the western United States (*1*). A major public health concern associated with wildfires is exposure to air pollutants, such as fine inhalable particles in smoke, with aerodynamic diameters ≤2.5 *μ*m (particulate matter [PM]_2.5_). Exposure to wildfire smoke can irritate the lungs, alter immune function, and increase susceptibility to respiratory infections. In addition, exacerbations of asthma, chronic obstructive pulmonary disease, cardiovascular disease, and possibly increased mortality are associated with smoke exposure* (*2*). Characterizing smoke exposure levels for communities located near the fires and personnel involved in response efforts is a critical public health function during wildfire episodes.

To characterize smoke exposure levels, CDC deployed two staff members to support the Interagency Wildland Fire Air Quality Response Program (IWFAQRP)^†^ as air resource advisors for wildfire incidents in California and Oregon. Air resource advisors are fully integrated into the wildfire incident management teams and provide insights into understanding and predicting air pollution exposures from wildfire smoke emissions. Air resource advisors interact with various stakeholders, including air quality regulators, fire personnel, public health practitioners, and community residents. A primary aspect of this engagement is to forecast smoke levels for areas immediately affected by fires and generate a daily smoke outlook^§^ for keeping stakeholders informed of prevailing smoke levels. 2020 is the first year during which CDC worked with IWFAQRP and deployed staff members as air resource advisors for wildfire incidents. From August 31 to September 14, 2020, one CDC staff member supported wildfire incidents in central Oregon’s Cascade Range, which included Beachie Creek, Holiday Farm, Lionshead, and Riverside wildfires. Strong east winds across the Cascade Mountains resulted in >560,000 acres of fire growth during September 7–10 (*1*). Another CDC staff member was deployed for the Creek fire incident from September 20 to October 5, 2020. The Creek fire incident in the Southern Sierra Nevada region of California started September 4 and grew to 193,000 acres during its first week (*1*); as of December 3, 2020, the fire had consumed 379,895 acres.^¶^

During these two deployments, several public health concerns came to light. Of note, although smoke from wildfires drifted long distances and affected downwind communities, the brunt of poor air quality was observed in communities adjacent to wildfire incidents. For example, communities located near the fire perimeter of wildfire incidents in California and Oregon experienced high concentrations of PM_2.5_, as measured by air quality monitors, resulting in “Unhealthy” to “Hazardous” conditions, as defined by the U.S. Environmental Protection Agency Air Quality Index** (Figure). Fire personnel who camped and rested between work shifts at nearby fire camps^††^ (North Fork, California and Sisters, Oregon) were also exposed to poor air quality levels. These fire camp exposures contribute to higher overall cumulative smoke exposure and, along with other occupational risk factors such as fatigue and stress, could limit recovery that is much needed for fire personnel while away from the active fire perimeter. In addition, environmental hazards such as extreme heat and higher concentrations of ambient carbon monoxide^§§^ were prevalent during days with heavy smoke and after extreme fire growth days; these hazards added a layer of complexity to fire response efforts and might have limited fire personnel recovery^¶¶^ between work shifts.

**FIGURE:**
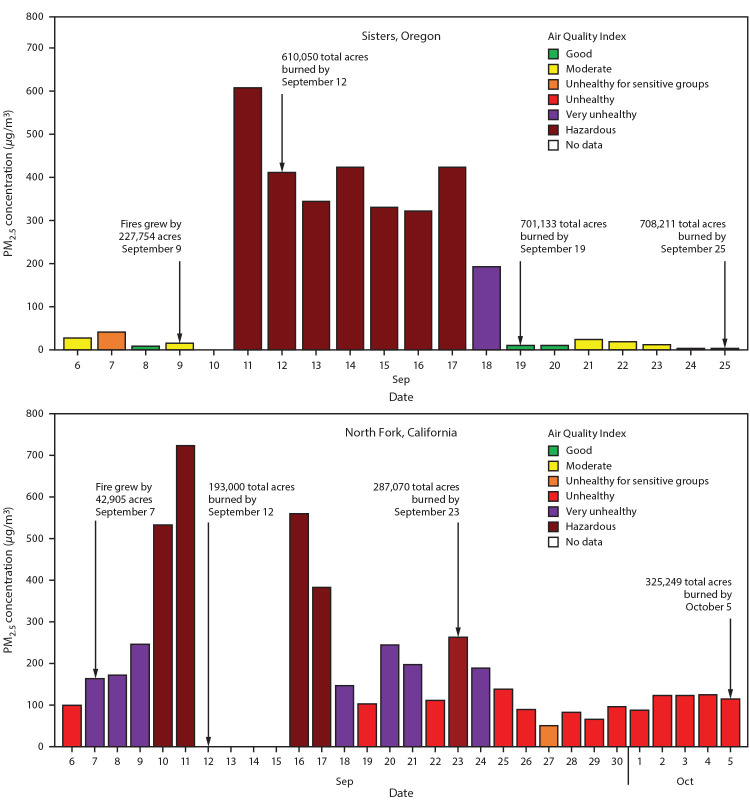
Daily 24-hour average particulate matter concentrations and Air Quality Index * for selected fire camp† sites during air resource advisor deployments — California^§^ and Oregon,^¶^ September–October 2020 **Abbreviation:** PM_2.5_ = particles with aerodynamic diameters ≤2.5 *μ*m. * Sensitive groups include persons aged ≤18 years; adults aged ≥65 years; pregnant women; persons with chronic health conditions such as heart or lung disease, including asthma and diabetes; outdoor workers; persons experiencing homelessness, and those with limited access to medical care. (https://www.cdc.gov/air/wildfire-smoke/default.htm). ^†^ Fire camps typically offer logistical support to the wildfire suppression operation by providing firefighters and incident personnel sleeping locations (camping), morning and evening meals, workspaces, and administrative services. ^§^ The monitoring instrument in North Fork, California, recorded errors and did not report data during September 12–15, 2020. ^¶^ Start date of Creek Fire in California was September 4. Start dates of fires in Oregon were as follows. Lionshead was August 16; Beachie Creek was August 16; Holiday Farm was September 7; Riverside was September 8.

High smoke levels and other hazards present during wildfires reinforce the need for professionals such as air resource advisors, who are trained in smoke-health issues and related public health topics, to be included on wildfire incident teams. Observations made by CDC staff members, as well as other air resource advisors, on environmental and occupational risk factors and their public health consequences on nearby communities and fire camps can inform future wildfire response efforts. Coordination among public health and land management agencies at multiple levels before, during, and after wildfire incidents can help mitigate adverse health effects. CDC continues to collaborate with federal land management agencies, which manage and support wildfire incidents, to understand the adverse health impacts of smoke exposure on communities and wildland firefighters.

## Footnotes

* https://www.cdc.gov/air/wildfire-smoke/default.htm.

† https://wildlandfiresmoke.net.

§ https://tools.airfire.org/ara/deployments/.

¶ https://inciweb.nwcg.gov/incident/7147.

** https://www.airnow.gov/aqi/aqi-basics/.

†† Fire camps typically provide logistical support to the wildfire suppression operation by providing firefighters and incident personnel sleeping locations (camping), morning and evening meals, workspaces, and administrative services.

§§ https://www.nasa.gov/feature/jpl/nasa-monitors-carbon-monoxide-from-california-wildfires.

¶¶ https://www.nifc.gov/PUBLICATIONS/redbook/2020/Chapter07.pdf.

## References

[1] National Interagency Coordination Center. Incident management situation report archives. Boise, ID: National Interagency Coordination Center; 2020. https://www.predictiveservices.nifc.gov/intelligence/archive/archive2020.html

[2] Reid CE, Brauer M, Johnston FH, Jerrett M, Balmes JR, Elliott CT. Critical review of health impacts of wildfire smoke exposure. Environ Health Perspect 2016;124:1334–43. 10.1289/ehp.1409277 27082891 PMC5010409

